# Dental trauma simulation training using four splinting models: A cross sectional study

**DOI:** 10.1111/edt.12772

**Published:** 2022-06-26

**Authors:** Sobia Zafar, Christine I. Peters

**Affiliations:** ^1^ School of Dentistry The University of Queensland Herston Queensland Australia

**Keywords:** avulsion, dental students, splinting, trauma simulation

## Abstract

**Background/Aim:**

Opportunities for dental students to obtain clinical experience in the management of traumatic dental injuries are scarce, and most dentists encounter difficulties with their first trauma patients after graduation. The aim of this study was to question students on the ease of handling of four types of flexible splints, with two common methods of bonding to the tooth.

**Material and methods:**

A total of 161 fourth year dental students completed a simulated treatment of an avulsed tooth using orthodontic wire, Twistflex wire, nylon fishing line, and Powermesh as splints. The bonding materials were composite resin (Spectra ST LV) or glass ionomer cement (GC Fuji LC Ortho). The students then answered 16 questions on a 5‐point Likert scale, or with an open answer field.

**Results:**

Most students agreed (48.8%) or strongly agreed (31.3%) that the simulated trauma exercise assisted their learning. There was strong agreement (68.8%) and agreement (28.7%) that the simulation added value to their dental training compared to didactic training only. Similarly, 52.3% of participants strongly agreed and 40% agreed that they felt engaged in the learning activity. Only 53.8% of the participants agreed and 7.5% strongly agreed that the simulation felt realistic. Most students (56.2%) found a Powermesh/composite splint was the easiest to place, and nylon fishing line/GC Fuji LC Ortho splints was the least difficult to remove (35%).

**Conclusion:**

Wire‐free splints with composite bonding were judged as the easiest to place by students, while glass ionomer cement was the easiest to remove.

## 1 INTRODUCTION

Dental traumatology is one of the emergencies dentists feel most insecure about and that dental students have limited exposure to before they graduate.^1^ The degree of knowledge regarding the management of dental injuries is not high among clinicians and a worldwide concerning lack of knowledge has created barriers to patient care. Nevertheless, dental trauma treatment is carried out regularly by dentists. This dilemma frequently results in inadequate emergency treatment of dental trauma and this may affect the prognosis of teeth after many dental injuries.^2^, ^3^, ^4^, ^5^, ^6^ In surveys on the emergency management of tooth avulsion, less than 50% of general dental practitioners followed the correct splinting strategy or duration of splinting.^7^, ^8^ Another survey showed that a considerable number of dentists were unsure whether to replant primary teeth or to use a rigid splint after tooth avulsion,^9^ and they apply treatment modalities that do not follow the contemporary standards of practice.

These findings highlight the need to improve the practical exposure of dental students to trauma situations in pre‐clinical and clinical settings.^1^ The University of Adelaide in Australia provides animal cadaver models for a realistic scenario and has incorporated the use of a sheep mandible for replantation and splinting training.^1^ Other institutions teach through simulated exercises involving 3D printed teeth.^10^, ^11^ In oral and maxillofacial surgery and dental education, 3D printed models are successfully used to demonstrate the effects of impact and to create new fractures.^12^

To avoid any damage to the enamel during removal after the splinting period, some authors recommend glass‐ionomer cement (GIC), such as GC Fuji Ortho LC (GC Australasia Dental Pty Ltd.), as an alternative to composite resin.^1^ Both GIC and composite resin are adherent materials that are easy to apply but composite resin is more time consuming and often complex to remove. GIC may be removed with hand instruments and its removal may cause less damage to the enamel of both the injured and supporting teeth.^13^

Splints may consist of flexible metal stainless steel wire, nickel titanium orthodontic wire, fishing line, fiber, titanium mesh, or composite resin only that is bonded to the injured and adjacent teeth with composite resin.^13^ Titanium Trauma Splints (Medartis AG), nylon splints, Ribbond (Ribbond Inc.), Kevlar, and fiberglass splints (EverStick, Stick Tech Ltd.) have been used with some resin materials proving too rigid after bonding.^14^ Other authors have described using more flexible materials such as Twistflex wire (3 M Unitek) and Power chain (Yancheng TC Medical Equipment Co., Ltd.).^15^ The Twistflex wire is constructed as an intertwined wire rope to form a single strand and is regularly used in orthodontics, for example, as a retainer and when maximum flexibility is desired.^16^ Power chain consists of flexible rubber loops and is used in orthodontics to exert a pulling force. It is inexpensive and similar in elasticity to nylon fishing line, while offering the advantage of ease of application, with chain openings that contain the composite material well, similar to the Titanium Trauma Splint.^15^ The most commonly recommended types of splints are orthodontic wire and fishing line, which are easier to place due to their ease of accessibility and low cost.^3^, ^11^ Procedural complexity influences the likelihood of a technique or material being used for the splinting method, and it is well known that the materials that are simple to use and methods that are simple to interpret are more likely to be adopted.^17^

A recent study by Zafar et al.^11^ used a 3D printed tooth model in a simulated avulsion scenario in a standard Typodont jaw model to evaluate the ease of application and removal of two types of splints (orthodontic wire with a composite resin splint and GIC with nylon fishing line). Two thirds of the participating students either did not favor or were neutral in their opinion toward splinting with a nylon fishing line/GC Fuji LC Ortho splint compared to a wire/composite resin splint. While nylon fishing line was judged as easier to use in other investigations, students in that study did not prefer it over orthodontic wire. However, more than half of the students noted that GIC was less cumbersome to remove than composite. In addition to easier handling, the nylon fishing line/GC Fuji LC Ortho splint was less damaging to the enamel during the removal process.

The aim of this study was to investigate the ease of procedural handling for student novices of four types of flexible splints, while using two common methods of bonding to the tooth. Participating fourth‐year students were invited to complete an anonymous questionnaire on the usefulness and realism of their experiences during the exercise. The null hypotheses were that there is (1) no statistically significant difference in the type and bonding method of the examined splints, and (2) the simulated trauma exercise was a valuable learning experience in trauma education for undergraduate dental students.

## 2 MATERIAL AND METHODS

Ethical approval for this study was obtained by the Institutional Human Research Ethics Committee (Approval number: 2019002969).

The design was a cross‐sectional study that followed the STROBE statement. The study involved completion of an anonymous questionnaire by 161 fourth‐year dental students enrolled in a Bachelor of Dental Science (Honours) degree at The University of Queensland, Australia. All fourth‐year students enrolled in DENT4070 (Advanced Dental Disciplines A) in Semester 1 (2021 and 2022) were invited to participate in the study and to answer questions on their experiences during simulated trauma splinting. The participant consent and information sheet, along with the questionnaire were distributed to students who agreed to participate. The students received a 60‐minute lecture on splinting prior to the simulation activity, which was preceded by a lecture series on dental trauma in their previous (third) year of studies (9 h in total). The simulation training took place in a 2‐h session, followed by a post‐training questionnaire.

**FIGURE 1:**
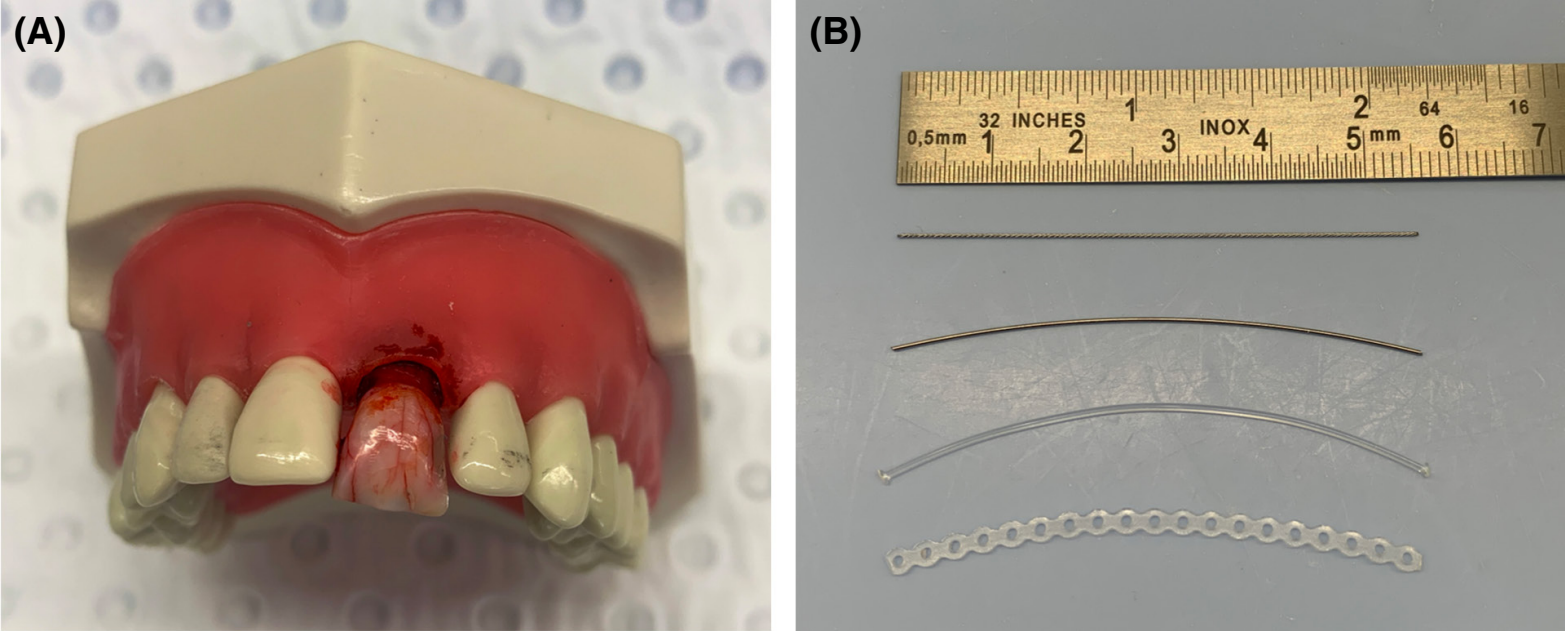
(A) Example of the avulsion model using a human central incisor, (B) Splinting materials used for the exercise from top to bottom: Twistflex wire, 0.4 mm of orthodontic wire, 40 lb nylon fishing line, Powerchain

Materials used for the splinting simulation exercise included a natural human incisor to simulate an avulsion injury, which was fitted in the Typodont model as described previously.^11^ Splint types included orthodontic wire (Remanium; Dentaurum), Twistflex wire (Dentaflex; Dentaurum), nylon fishing line (Spectra Brutal Strong; Isorline), and Powermesh (Dynalink Elastomeric Chain; GH Orthodontics for Henry Schein). All students completed two composite resin splints using etch and bond (Super‐Etch Gel; Henry Schein) and composite resin (Spectra ST LV; Dentsply Sirona) and two GC Fuji LC Ortho splints LC (GC Australasia Dental Pty Ltd.). The material combinations were wire/composite resin splint, Powerchain/composite resin splint, nylon fishing line/GC Fuji LC Ortho, and Twistflex wire/GC Fuji LC Ortho. The model and types of splints used in the study are shown in Figure 1.

**FIGURE 2:**
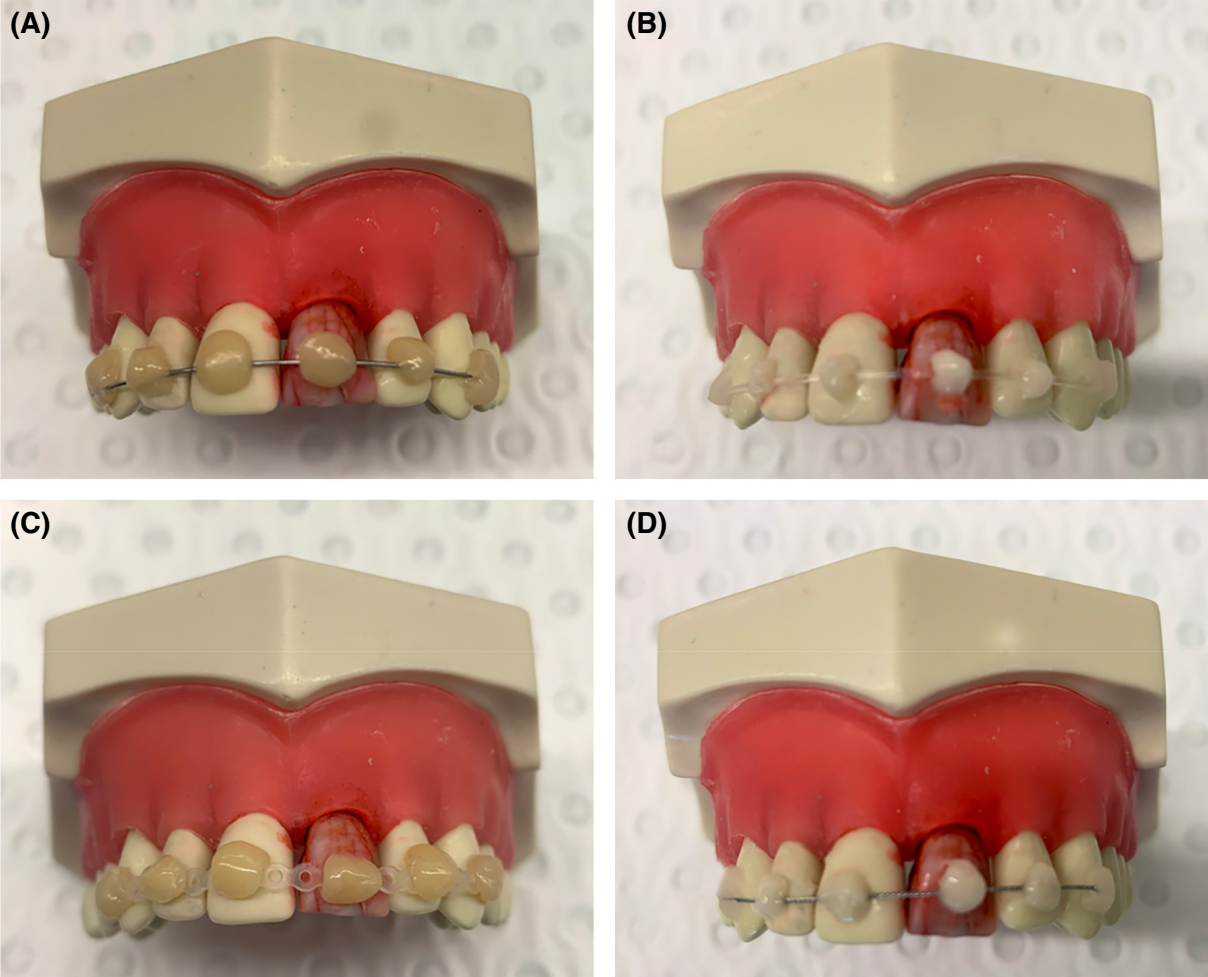
Splinting material combinations used in the pre‐clinical exercise. The material combinations were (A) wire/composite resin, (B) nylon fishing line/GC Fuji LC Ortho (C) Powerchain/composite resin, and (D) Twistflex wire/GC Fuji LC Ortho

Students received stepwise illustrated instructions before the scheduled session and they watched live demonstrations by a tutor before commencement of the exercise. Working in pairs, each student alternated with their partner in carrying out the role of the dentist or the dental assistant for all exercises. For composite resin splints, students were instructed to prebend the wire (0.4 mm orthodontic wire, 0.15‐inch Twistflex wire) to fit flush on the labial surfaces of the teeth and to ensure the wire was attached to two teeth on each side of the correctly positioned avulsed tooth. The length of the wire extended two thirds toward the distal end of these teeth to avoid any sharp edges. Similarly, Powerchain was applied on the labial surface of the teeth for the second exercise. After etching the tooth surface and applying the bonding agent, the splint was positioned flat and fixed in place with composite resin using the Powerchain loops for retention. The splints were bonded to the teeth first and cured for 30 seconds, followed one by one by the avulsed tooth and lastly the adjacent teeth for both exercises. For the GC Fuji Ortho LC splints, students received similar instructions for the nylon fishing line, Twistflex wire, and GC Fuji Ortho LC splint. GC Fuji Ortho LC was syringed from the capsule onto the labial surfaces of the uninjured adjacent teeth, and the nylon fishing line or Twistflex wire was applied to the unpolymerized material and then adapted using a water moistened cotton pellet to cover the fishing line or Twistflex wire, and then it was partially cured for 10 s. The GC Fuji Ortho LC was applied to the labial surface of the correctly positioned avulsed tooth and then the entire splint was light cured for 40 seconds. All splint designs used in this study are shown in Figure 2. All four splints were subsequently removed with a flat plastic instrument or a spoon excavator.

The study utilized the previously validated simulation survey tool developed and used in an earlier study.^11^ Briefly, this was a 16‐item questionnaire consisting of a combination of multiple choice five‐point Likert scale questions, ranging from “strongly agree” to “strongly disagree,” and open‐ended question fields. The estimated completion time for the questionnaire was 10 min. The survey was administered once the students had completed both exercises in the pre‐clinical setting.

The data were tabulated in a Microsoft Excel (Version 16.30, Microsoft Corp.) spreadsheet and then imported into IBM SPSS Statistics for Macintosh v26 (IBM) for descriptive analysis and GraphPad PRISM 9.0 software (GraphPad Software) for collation and creation of appropriate graphs. Responses were summarized, and comparisons were made. Specific data analysis tests performed included descriptive statistics, such as frequencies and percentages.

## 3 RESULTS

A total of 165 students were invited to participate in the study, of which 161 agreed to take part and completed the questionnaire with a response rate of 95.2%. Of the final number of participants, 82 were female and 79 were male. The age of participants ranged from 20 to 39 years, with a median age of 22 years. A total of 97.5% of participants had no previous experience in dental trauma management and 2.5% had previous experience of observation in a clinical environment. Those with previous trauma experience perceived themselves as *“somewhat confident*.” The students' experience after the trauma simulation training was assessed through a series of statements to which students indicated their agreement (Table 1). The results of the questionnaire showed that majority of the students agreed (48.8%) or strongly agreed (31.3%) that this training assisted their learning. Similarly, 52.3% of the participants strongly agreed and 40% agreed that they felt engaged in the learning activity. However, only half of the participants (53.8%) agreed and 7.5% strongly agreed with the statement that the dental trauma simulation felt realistic. Excluding two participants, the remaining participants either agreed (28.7%) or strongly agreed (68.8%) with the statement that the use of simulation added value to their training compared to relying solely on traditional didactic training. Overall, 96.2% of participants agreed/strongly agreed that the simulation training should be a mandatory component of the dental curriculum (Table 1).

**TABLE 1:** Post trauma simulation training perception of dental student

Statement	Responses (%)				
	Strongly agree	Agree	Neutral	Disagree	Strongly disagree
The dental trauma simulation felt realistic	6.0 (7.5)	43 (53.8)	17 (21.3)	10 (12.5)	3 (3.8)
The dental trauma simulation training assisted my learning	38 (47.5)	39 (48.8)	1.0 (1.3)	1.0 (1.3)	1 (1.3)
By practicing in the simulation, I feel I am more prepared for working on dental patients with traumatic injury	25 (31.3)	46 (57.5)	6.0 (7.5)	2.0 (2.5)	1 (1.3)
The use of simulation added value in my training compared to relying solely on traditional didactic training	55 (68.8)	23 (28.7)	0 (0)	0 (0)	2 (2.5)
I felt engaged in the learning activity	42 (52.5)	32 (40)	5 (6.3)	0 (0)	1 (1.3)
Dental trauma training in the simulation clinic should be a mandatory part of the curriculum	54 (67.5)	23 (28.7)	1 (1.3)	0 (0)	2 (2.5)
The instruction material including lectures, hand‐outs, and models was of a high standard	34 (42.5)	43 (53.8)	1 (1.3)	0 (0)	2 (2.5)

The comments of the students regarding realism included “*would like to observe real‐life cases*”, “*would like to gain experience in primary tooth trauma management*,” “*demonstration of real trauma management videos in children*” and “*demonstration of the behaviour management techniques in a child during dental trauma scenarios*.” In response to the question about what would they like to be included in a trauma simulation, the participants requested “*additional splinting exercises for subluxation injuries*,” “*more training with other splinting materials*,” and “*more opportunities to work with GC Fuji LC Ortho*.”

**FIGURE 3:**
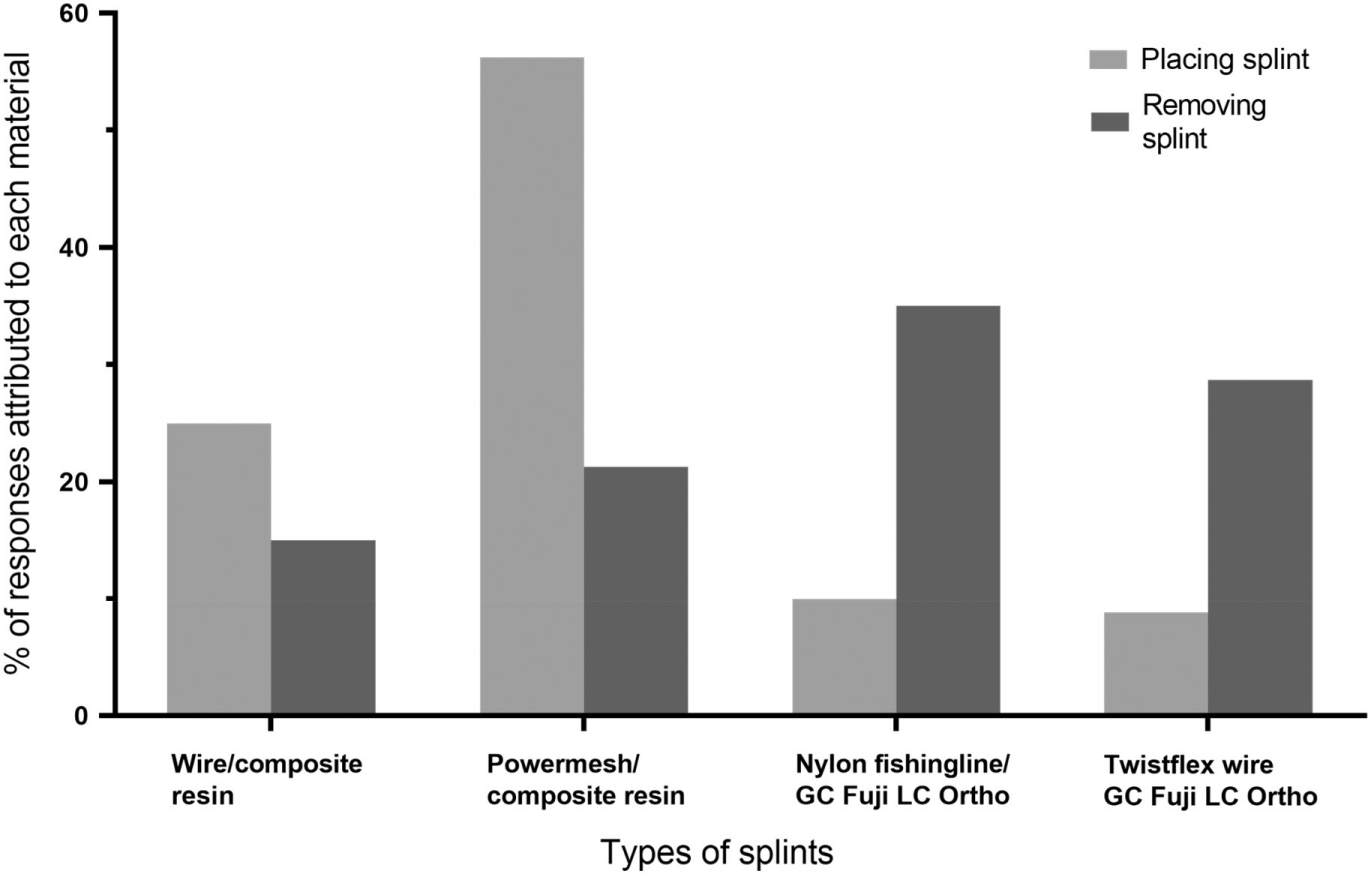
Participants rating of the ease of placement and removal of the various splints

After the use of the splinting materials, 56.3% of the participants rated the Powermesh/composite resin splint as the easiest to apply, followed by 25% for wire/composite resin, 10% for nylon fishing line/GC Fuji LC Ortho, and 8.8% for Twistflex wire/GC Fuji LC Ortho. Concerning ease of removal, nylon fishing line/GC Fuji LC Ortho was the highest rated with 35% judging it as easy to remove, followed by 28.7% for Twistflex wire/GC Fuji LC Ortho, 21% for Powermesh/composite, and 15% for wire/composite resin (Figure 3). When asked what difficulties they encountered in conducting the trauma simulation exercise, the participants' responses included: “*manipulating GIC and holding wire in place*,” “*taking off composite*,” and “*the application and setting time for Fuji Ortho was not ideal*.”

## 4 DISCUSSION

Simulated trauma splinting exercises are an important part of preclinical training for undergraduate dental students. Often dental students lack clinical hands‐on exposure to trauma management in patients before graduation.^8^ Later in their career, dentists perceive barriers to effectively treating dental trauma patients, and aside from time constraints and uncooperative patients, the lack of knowledge and skills are cited as obstacles.^9^, ^18^ The results of this study showed that the first null hypothesis that no statistically significant difference in type and bonding method of the examined splints must be rejected, as wire‐free splints were easier to place than wire splints and composite resin was more difficult to remove than GIC. Part 2 of the null hypothesis was confirmed in that the simulated trauma exercise added a valuable learning experience in trauma education for undergraduate dental students.

Clinician competence is being scrutinized, with demands made of proof of continuing education, and such inspection affects both undergraduate dental student teaching as well as upskilling of dental professionals.^19^, ^20^ Trauma treatment guidelines delineate how treatment can be rendered in a timely and efficient manner, avoiding lengthy exposure or manipulation of the injured site.^3^, ^4^, ^5^ Choosing appropriate materials and techniques influences healing results, as well as patient comfort in the areas of sensitivity of traumatized and splinted teeth, gingival inflammation, lip abrasions, speech impediments, food intake, and the ability to maintain oral hygiene.^21^ Effective pre‐clinical training may help counteract the reality that the information in published trauma guidelines may not reach every dental clinician.^22^

Literature on dental student trauma exercises supports both GIC and composite resin as satisfactory bonding materials for splints.^11^ Nevertheless, it is unclear which type of splint is preferred by learners. The aim of this study was to examine the ease of handling of different splint types for student novices in simulated trauma exercise, while employing two accepted methods of bonding in an avulsed tooth scenario. An anonymous survey completed by the students brought answers on effectiveness and practicality of their experiences during the exercise.

In the learning task investigated in this study, an avulsed tooth scenario using a natural human maxillary incisor with blood (red paint) was employed. The dental students replanted the incisor tooth into a Typodont socket to simulate a recent avulsion injury. This model has opened new avenues for training specific dental treatments that are difficult to replicate in a pre‐clinical setting.^10^ The materials used were orthodontic wire and Twistflex wire as flexible metal splints and Powerchain and nylon fishing line as non‐metal splints. The two types of wire are promoted as suitable in the trauma literature. Composite resin and wire splints may be the most commonly used flexible splints in clinical practice.^13^ The wire diameter should be no greater than 0.016 inch or 0.4 mm, which was the case for both types of wire selected.^3^ Powerchain and nylon fishing line are non‐metal alternatives to wires and are comparable in flexibility.^15^ Powerchain is a flexible, aesthetic, low‐cost alternative to nylon fishing line that is used as in wire‐free intermaxillary fixation in fractures of the jaws.^23^ Powerchain was integrated into the study for its reported ease of adaptation, with negligible problems during application.^15^

In this study, one wire and one wire‐free splint each was fixed with GIC or composite resin. The glass ionomer selected is commonly used in orthodontic bracket bonding and the etched and bonded composite resin was a material used for direct restorations in pre‐clinical courses. After completion of the splinting tasks as either operator or assistant, students filled in a voluntary, anonymous questionnaire. In a pre‐clinical setting, realistic, extensive, and repetitive hands‐on training is needed to prepare students for their first real‐life patient.^10^ In this investigation, 80.1% of the participating dental students agreed or strongly agreed that the training exercise assisted their learning and only two students had previously observed a trauma treatment in a clinical setting. Except for two students, all those surveyed agreed or strongly agreed that the use of simulation brought added value to their pre‐clinical education in addition to traditional didactic training. This aligns with reports by authors who investigated 3D printed models in training final year undergraduate students in Munich, Germany.^10^ Their model was based on a CBCT of a real patient, accompanied by a realistic clinical scenario, and 97% felt better prepared for treating traumatic dental injuries in the future after undergoing trauma training.

A high proportion (92.3%) of students found the learning activity interesting, but only 53.8% agreed and 7.5% strongly agreed that the simulated dental trauma felt realistic enough. Student remarks in an open answer field included a wish for more clinical scenarios and inclusion of different types of luxation injuries. This area could be improved in future trauma simulations, for example, by offering a patient case scenario based on a real patient as suggested by other authors, with diagnosis and treatment planning alongside the practical component, as well as incorporating a possible selection of traumatic injuries.^24^ Overall, a majority of 96.2% of students who completed the trauma splinting exercise agreed or strongly agreed that the simulation training should become mandatory in the dental curriculum. Considering that the likelihood of dental students achieving hands on exposure to real life trauma remains rare, and immediate emergency management of a stressful dental injury by experienced clinicians is preferred, knowledge of new dentists in dental trauma treatment will continue to be limited.^25^ In a questionnaire regarding dental trauma treatment strategies, two‐thirds of the queried dentists felt unprepared in treating an avulsed tooth.^26^ Apart from attending continuing education courses, simulated laboratory scenarios are a valuable adjunct in practicing components of the treatment, such as the repositioning of an avulsed tooth and bonding a correctly designed splint.

Treatment of traumatic dental injuries in permanent teeth expediently can strongly impact the tooth's prognosis.^2^ Methods that are uncomplicated and quick can help in rendering timely care. When placing trauma splints, most students (56.3%) found that a Powermesh/composite resin splint was the easiest to apply, followed by wire/composite resin and there were similar values in the ease of application for nylon fishing line/GC Fuji LC Ortho and Twistflex wire/GC Fuji LC Ortho. Judging composite resin as the least cumbersome option of bonding is somewhat surprising in that the glass ionomer cement does not involve the steps of etching, rinsing, drying, bonding, and light curing the monomer as well as the material. Students, however, remarked that “manipulating GIC and holding the wire in place” was difficult and that the applied material set faster than expected. The study by Hassan et al. confirms that Powermesh lies passively on the tooth and the shape of the connected loops makes composite easy to apply, while wires such as Twistflex wire were difficult to bend into the right shape and they tended to slip during application.^15^ The participating students were familiar with composite resin from their restorative dental training and they had had no previous exposure to GC Fuji LC Ortho, which could explain the feeling of being less comfortable with the new material. Removal of splints was felt to be more problematic than the application, especially when composite resin was used. Regarding splint removal, 35% of the students judged nylon fishing line/GC Fuji LC Ortho as the easiest to remove, followed by Twistflex wire/GC Fuji LC Ortho, Powermesh/composite resin, and wire/composite resin. Students stated that difficulties were encountered in *“taking off composite,”* and this is corroborated in the literature. Composite resin is more time‐consuming to remove and avoiding any damage to the enamel is challenging.^1^, ^13^, ^14^ In the case of a traumatic dental injury, timely and up to date care is required to avoid functional or aesthetic problems for the patient. Notwithstanding this, general dentists often lack knowledge in rendering appropriate care for a traumatized tooth.^7^, ^20^, ^22^, ^26^

One of the limitations of the study was that Typodont models do not replicate the physical and chemical composition of enamel which is crucial for this pre‐clinical trauma exercise. Additionally, extracted human teeth do not behave the same as natural teeth in the patient's mouth, and there was no impact of any patient‐related factors, for example, a distressed child, saliva, and tongue movements. Thus, this may have impacted the students' perception of the application and ease of splint removal. Future recommendations include investigating whether the TDI management skills that were learned in the workshop are transferred to the clinic.

## 5 CONCLUSIONS

Adding simulated trauma exercises to the dental curriculum enhanced education for participating undergraduate students. It provided valuable learning benefits while stimulating interest to a degree that most students felt the training should become mandatory. Of the splints used, wire‐free Powerchain and nylon fishing line were easier to apply compared to orthodontic wire or Twistflex wire, while glass ionomer cement was easier to remove than composite resin.

## 6 AUTHOR CONTRIBUTIONS

Sobia Zafar and Christine Peters: conceptualisation, Methodology, Writing, Editing, Review, Project Adminstration.

## CONFLICT OF INTEREST

The authors declare no conflict of interest.

## Data Availability

The data that support the findings of this study are available from the corresponding author upon reasonable request.
